# The investigation of parameters affecting Ibrutinib release from chitosan/tripolyphosphate/carbon nanofiber composite microspheres

**DOI:** 10.55730/1300-0527.3466

**Published:** 2022-07-19

**Authors:** Canan ARMUTCU

**Affiliations:** Department of Chemistry, Faculty of Science, Hacettepe University, Ankara, Turkey

**Keywords:** Chitosan, carbon nanofiber, composite, Ibrutinib, controlled drug delivery

## Abstract

This study described the performance of carbon nanofiber modified chitosan (CNF@CS) composite microspheres for the controlled release of the Ibrutinib (IBR) drug. The surface morphology, particle sizes, and functional group contents of the microspheres were characterized by attenuated total reflection-Fourier transform infrared spectroscopy (ATR-FTIR), scanning electron, and optical microscopy measurements. The obtained data demonstrated that the addition of CNF to the microsphere increased the encapsulation efficiency of the IBR while allowing the controlled and gradual release of the drug. In terms of the encapsulation efficiency and drug release rate, IBR@CS/TPP/CNF microspheres, achieving drug encapsulation efficiency of 83.09%, have the most suitable formulation according to the comparative studies. Furthermore, according to Korsmeyer-Peppas kinetic model, IBR release mechanism was anomalous diffusion (swelling-controlled behavior and diffusion.) because the IBR release profile was completed in 78 h under optimized conditions. Therefore, the development of CNF based chitosan microsphere is a promising approach to assure appropriate dosage, safety, and improving drug efficacy.

## 1. Introduction

Controlled drug delivery is a multidisciplinary field that aims to achieve the desired clinical response in biological organisms in the most effective way. Reducing excess chemical exposure protects the patient from side effects arising from overdose and prevents early elimination of the drug by directing it to the targeted area. Drug delivery systems can have a broad spectrum depending on the treatment of interest and successfully take place in the literature as an influencing method for intensive drug treatments such as chemotherapy [1].

Ibrutinib (IBR), a small molecule chemotherapeutic that is used as irreversible inhibitor of Bruton’s tyrosine kinase (BTK), and acts as a promising drug on several B-cell malignancies [2,3]. IBR is an approved drug for the treatment of mantle cell lymphoma (MCL) and chronic lymphocytic leukemia (CCL) in the past 10 years [4]. IBR is mainly absorbed very rapidly by the small intestine, mainly. Because IBR has poor solubility in water, its extremely low oral bioavailability causes high dosage applications. As with most oral drugs, taking IBR in higher doses causes drug toxicity and undesired side effects [5,6]. To overcome these problems, microspheres have received much attention to design efficient carrier materials for controlled or sustained drug release. Microspheres are prepared using carbon-based materials such as carbon nanofibers, carbon nanotubes, and graphene oxide for enhancing drug-loading capacity mechanical strength and release drugs at desired times [7–9].

Carbon nanofiber (CNF), having special cylindrical or conical one-dimensional (1D) nanostructures, provide good dispersibility and operability [10]. CNF is a potential candidate to increase the drug encapsulation efficiency, provide mechanical strength, and sustained controlled release of the drug [11]. Moreover, the ability to make strong electrostatic interactions with many adsorbates, low toxicity, high surface area, and flexibility allow CNF to combine various alternative biomaterials, including chitosan.

Chitosan (CS) is a natural cationic polysaccharide which is obtained by deacetylation of chitin [12]. This natural polymer has numerous important features such as excellent biocompatibility, biodegradability, ease-to-functionalize, and polycationic character. These advantages provide several pharmaceutical applications to develop effective drug delivery systems [13,14]. Chitosan microspheres can be prepared by many different approaches such as emulsification-solvent evaporation, coacervation, spray drying, and ionotropic gelation [4,15–17]. However, chitosan microsphere prepared by chemical crosslinker agent (usually glutaraldehyde), may occur their toxic reaction and undesirable effects. To solve this problem, tripolyphosphate (TPP) is widely used as a nontoxic polyanion that can interact with chitosan via electrostatic forces [14,16].

IBR controlled release studies with different materials are available in the literature [4,18]. For example, Zhao et al. prepared IBR-loaded nanoparticles based on chitosan and sulfobutylether-b-cyclodextrin (b-CD) and CS for IBR delivery. The results showed that nanoparticles have displayed high the encapsulation efficiency (76.9%) with increasing SBE-b-CD concentration [4]. Prasad et al. used Capryol 90 (oil), Cremophor RH40 (surfactant), and Transcutol P (cosurfactant) to synthesize self-nanoemulsifying drug delivery system (SNEDDS) in the delivery of Ibrutinib by oral route. The release studies were carried out pH 6.8 and the IBR release was achieved 83.78% in 15 min [18]. Herein, I used chitosan as the main material of the microspheres, and TPP as the crosslinker. Chitosan-TPP microspheres have a biodegradable natural structure while providing protection from undesirable toxic effects. Moreover, the addition of CNF to the structure during the synthesis of microspheres increased the efficiency of drug encapsulation and provide the sustained controlled release of the drug. As far as I know, the prepared materials, which displayed highly drug encapsulation efficiency of IBR, has not been reported previously.

In this study, a new carbon nanofiber incorporated chitosan-based composite drug carrier was developed as an alternative system while using TPP as a crosslinker. CS/TPP/CNF composite microspheres were synthesized by ionotropic gelation method whereas the effecting parameters such as chitosan concentration, pH of TPP solutions, TPP concentration, IBR concentration, CNF concentration and crosslinking time were comparatively investigated. The releasing profiles of IBR from various CS/TPP/CNF composite microspheres were investigated to assure the safety and appropriate dosage of the drug. In addition, this study offers and proves that CNF incorporated composite microspheres have a high potential for releasing IBR in a sustained, controlled manner.

## 2. Materials and methods

### 2.1. Chemicals

Ibrutinib active ingredient was provided by TOBIO pharmaceuticals. Chitosan (medium molecular weight, and degree of deacetylation ≥85%) and sodium tripolyphosphate (NaTPP, technical grade 85%) were supplied from Sigma-Aldrich. Graphitized carbon nanofibers (200–600 nm outside diameter, 5–50 mm length) were supplied by Grafen Chemical Industries Co. Sulphuric acid (H_2_SO_4_), nitric acid (HNO_3_), methanol (MeOH), and acetic acid (HAc) were of analytical grade and obtained from Merck.

### 2.2. Preparation of composite microspheres

**Figure 1:**
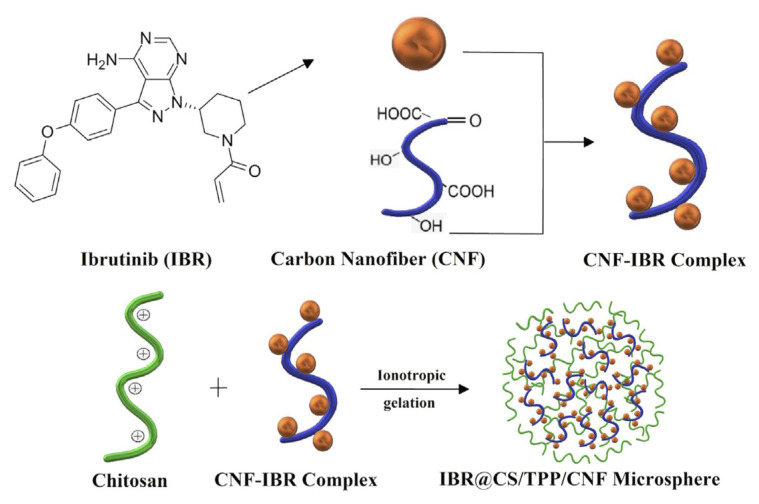
Schematic of the formation of IBR@CS/TPP/CNF microsphere.

Chitosan/Tripolyphosphate/Carbon nanofiber (CS/TPP/CNF) microspheres containing Ibrutinib were prepared by an ionotropic gelation procedure (Figure 1) [19]. For carbon nanofiber oxidation: the specific amounts of CNF powder were dispersed in 8.0 mL, 4.0 M aqueous mixture of H_2_SO_4_/HNO_3_ (3/1, v/v) and treated at 70 °C for 180 min [20]. The treated CNF powder was placed in a beaker and washed with deionized (DI) water until the pH value of the washing-out solution was equal to 7.0. Washing removes O-bonded oxides to the carbon nanofiber, so directly N- and S- bonded oxides are retained [21]. Then, the sample was dried at 70 °C for 48 h.

Herein, carbon nanofibers (CNFs) were treated with IBR in order to achieve proper drug loading to composite microspheres. In this respect, a desired amount of IBR was added into 1.0 mL MeOH-deionized water solution and subsequently magnetically stirred at 100 rpm at 25 °C for 2 h. Afterward, the CNF (0.05%, 0.1%, 0.2% w/v) was added into the solution and stirred for 4 h to obtain homogeneous complex dispersion. Ibrutinib and Ibrutinib-CNF complexes were dispersed in chitosan solutions in (2% v/v acetic acid) until homogenized dispersion was obtained. The composite microspheres were formed by dropping via peristaltic pump (1.0 mL/min, flow rate) into TPP solution. The gelled composite microspheres were collected and washed several times by DI water. Finally, IBR@CS/TPP/CNF microspheres were isolated by filtration and lyophilized at –50 ^o^C against 1.0 mmbar pressure for 48 h.

In this study, the effect of chitosan concentration (1.5%, 2%, 2.5% w/v), pH of TPP solutions (pH 5.0, pH 7.0, and pH 8.8), TPP concentration (1%, 2%, 3% w/v), IBR concentration (10%, 20% and 25% w/w), CNF concentration (0.05%, 0.1%, 0.2% w/v), and crosslinking time (30, 60, and 120 min) were investigated for optimization of IBR@ CS/TPP/CNF microsphere properties (Table 1).

### 2.3. Structural analyses of microspheres

The surface morphology of the composite microspheres was studied by scanning electron microscopy (SEM) (GAIA3, Tescan, Brno, Czech Republic). The lyophilized microspheres were coated with a gold layer for enhancing conductivity. Attenuated total reflection-Fourier transform infrared (ATR-FTIR) spectrophotometer (Diffuse Reflectance, Thermo model-NICOLET-IS 10 FTIR) was conducted to characterize the functional groups of microspheres. The ATR-FTIR spectra of the composite microspheres were recorded from 4000 to 500 cm^−1^. Particle sizes were measured using an optical microscope fitted with a micrometer by which the size of the microspheres could be determined. In order to calculate the average size properly, fifty composite microspheres of all formulations were used in these measurements [19].

### 2.4. Determination of IBR encapsulation efficiency

IBR encapsulation efficiency was measured by dividing the trapped IBR amount in the microspheres by the total amount of IBR added into the chitosan solution. The residual IBR in the TPP gelling and washing solutions was also collected and measured by UV/Vis spectrophotometer (Shimadzu, UV-1280, Tokyo, Japan) at 260 nm. The drug encapsulation efficiency of microspheres was calculated by using the following equation:


(1) $$\text{Encapsulation efficiency }(\text{EE})=\frac{\text{total IBR-residual IBR}}{\text{total IBR}}$$

All samples were repeated thrice while the average values were reported.

### 2.5. IBR release studies

The IBR releasing profiles of the composite microspheres were investigated in physiologic pH value. Briefly, 30 mg of the composite microspheres were placed in 4.0 mL of buffer solutions (pH 6.8) at 37 ± 0.5°C and were placed on the shaking incubator at 100 rpm (JSSB-30T, JSR, Gongju, Korea). IBR is a weakly basic drug whose solubility depends on pH. It is soluble in acidic pH conditions, but weakly soluble in neutral pH conditions [22]. Furthermore, IBR is mainly absorbed in the small intestine, thus the medium was adjusted to the simulated intestinal fluid (SIF, pH 6.8) to prevent the collapse of Ibrutinib drug and to realize drug release effectively [4, 23]. In the predetermined time intervals, 0.4 mL of the buffer solution was sampled and replaced with 0.4 mL of the fresh buffer solution to keep the volume of the release medium constant. As a result, the amount of the released IBR was determined at 260 nm spectrophotometrically. All IBR release studies were performed in triplicate to calculate the standard deviation.

### 2.6. IBR release kinetics

To understand the release mechanism of IBR from the composite microspheres, the release data were analyzed using three standard kinetic models; zero-order (Eq. 2), first-order (Eq. 3), and Korsmeyer-Peppas (Eq. 4) models.


(2) $$\text{Zero-order model:}q_{t}=q_{0}+k_{0}\text{t}$$

(3) $$\text{First-order model:}In\;q_{t}=In\;q_{0}-k_{1}\text{t}$$

(4) $$\text{Korsmeyer-Peppas }{\text{model:M}}_{\text{t}}/{\text{M}}_{\infty }={\text{kt}}^{\text{n}}$$

The zero-order kinetic model is associated with a constant drug release rate, which is independent of the drug concentration. The first-order kinetic model identified with the rate of release which is linearly dependent on its drug concentration. Korsmeyer-Peppas model explains drug release from swellable polymeric systems, and drug release should be one-dimensional. For the general equation to be used, the M_t_/M_∞_ ratio must be less than 0.6 [24]. The determination coefficient (R^2^) in each kinetic model was calculated after fitting the pure data to each equation.

For the Korsmeyer-Peppas model; M_t_ means the amount of drug release at time t, M_∞_ describes the amount of drug release at an infinite time, k is the kinetic constant. The diffusional release exponent is n which is related to the mechanism of drug release. In the case of Fickian release (diffusional controlled-release), n should be close to 0.43, and when exactly equal to 0.43 [25]. The case II release occurs transport or swelling controlled, and n is 0.85. In the non-Fickian release, the exponent of n is approximately 0.43 to 0.85, and the system is both diffusion and swelling controlled [26].

## 3. Results and discussion

### 3.1. Structural analysis

**Figure 2:**
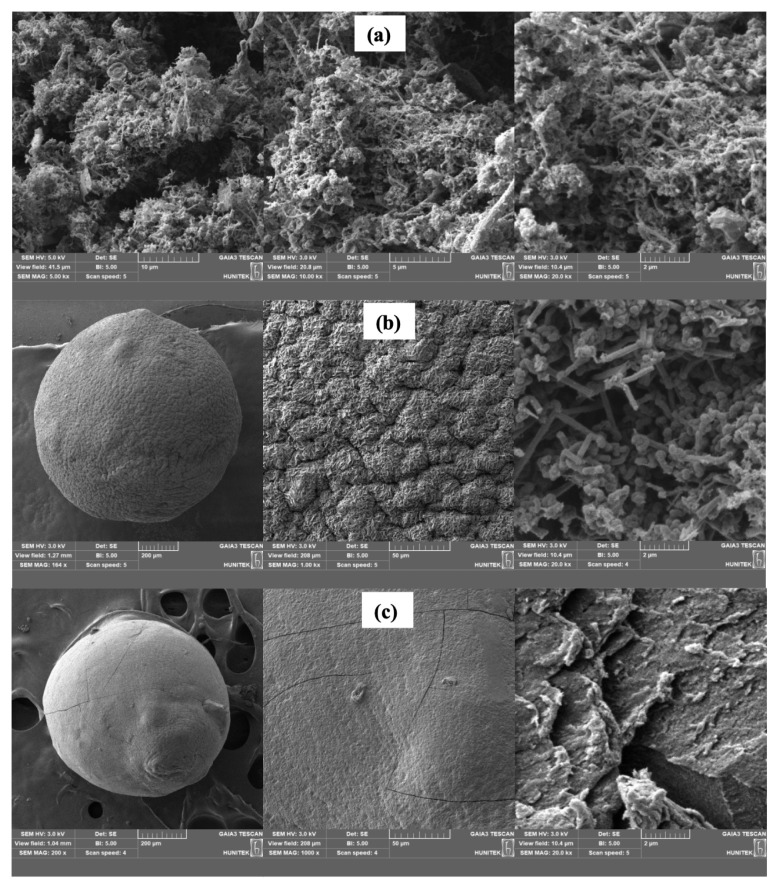
SEM images of CNF (a); C5-coded composite microsphere (b) and C15 microsphere (c).

Figure 2 shows the SEM images of the CNF sample, C5, and C15 microspheres. CNFs were formed a tight network that looks like tangled and hollow ropes (Figure 2a) [27]. Figure 2b indicates that the surface of C5 composite microspheres was quite smooth. However, the presence of CNF in the C5 composite microspheres changed the surface of microspheres to make the surface more wrinkled and rough. Moreover, adding CNF into chitosan resulted in folded chitosan microsphere surface [16]. To explain briefly, C5 composite microspheres were composed with the electrostatic interactions between negatively charged oxide CNFs and positively charged chitosan. It reduced the van der Waals forces among CNF clusters by wrapping the CNFs in the polymer structure, so a crinkly rough structure was formed on the surface [28]. Besides, from the SEM image of chitosan microsphere, it was clear that CNFs were trapped inside internal texture of microsphere which prevents the diffusing of the drug easily (Figure 2b), while IBR did not encounter hardship in release from the chitosan microspheres without CNF (Figure 2c).

**Figure 3:**
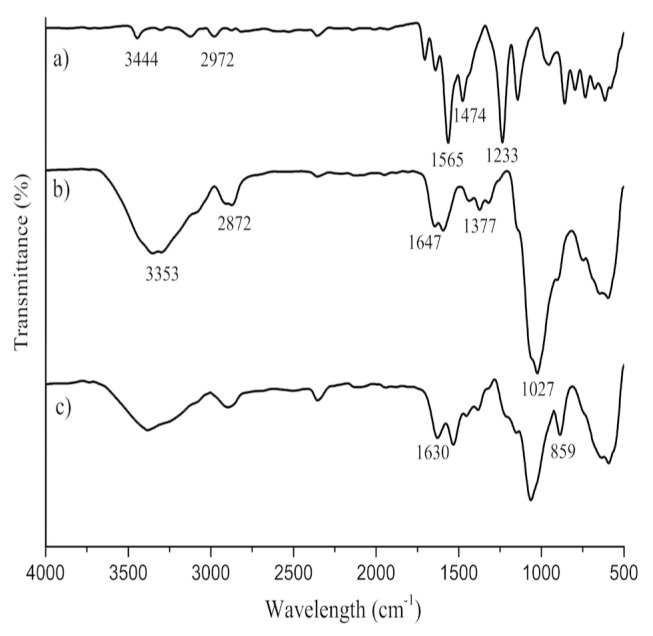
ATR-FTIR spectra of IBR (a), chitosan (b), C15 microsphere (c).

Functional groups of IBR, bare chitosan, and C5-coded composite microsphere were determined using ATR-FTIR spectrometer (Figure 3). The main characteristic bands of IBR (Figure 3a) were observed for -N-H stretching peaks at 3444 cm^−1^ and, for C-H stretching peaks at 2972 cm^−1^. Moreover, the bands at the 1565, 1474, and 1233 cm^−1^ were attributed C=C–C stretching, C=N stretching, and C-N stretching of an aromatic ring found in IBR structures. The broad peak at 3353 cm^−1^ corresponds to O-H groups vibrations in the chitosan spectrum (Figure 3b). The bands at 2872 cm^−1^ corresponded to stretching vibrations connected with the C−H bond. The peaks at 1647 and 1377 cm^−1^ belong to N-H and C-N stretching vibrations, respectively. The bands at 1027 cm^−1^ is due to the vibration of –C–O–C– groups. According to the spectra for C5-coded composite microsphere, the peak at 1630 cm^−1^ is ascribed to the stretching band of C=O in the structure of carbon nanofibers (Figure 3c). The characteristic peaks of IBR disappeared in the C5 microsphere due to the overlap of bands in this region comprising the deformation of -OH, -NH, and -CH_x_ groups. This situation can be explained due to the small IBR ratio [4] while the bands at 859 cm^−1^ indicate the present of the aromatic group (CH=CH) of IBR on the surface of CNF.

The particle size distribution of the microspheres varied between 0.58 and 1.16 mm (Table 2). The mean particle size increased with increasing the amount of chitosan, which also increased the viscosity of the polymer concentration [19]. In addition, the increase in TPP concentration [19] and crosslinking time [29] caused a further increased in the particle size of the microspheres, as expected.

### 3.2. Drug entrapment efficiency

Encapsulation efficiencies of IBR ranged between 17.80% and 87.55% in the formulations. The encapsulation efficiencies of C1, C2, and C3-coded composite microspheres were calculated as 49.89%, 31.55%, and 17.80%, respectively. When the pH of TPP solutions increased, the solubility of IBR increased in the external phase so the encapsulation efficiency was considerably reduced [29–31]. Considering the encapsulation efficiency, the pH of the TPP solution was kept constant at 5.0 in all formulations to retain the drug within the composite microspheres, and encapsulation efficiency significantly increased with the CNF content in synthesized composite microspheres. Electrostatic interactions between oxygenated functional groups of CNFs and IBR, together with hydrogen bonds, are the main reason for the increase in encapsulation efficiency. The studies suggested that formulations with higher ratios of chitosan, CNFs, and TPP improved the drug entrapment efficiency and decreased with increasing crosslinking time. (Table 3).

### 3.3. IBR release studies

**Figure 4:**
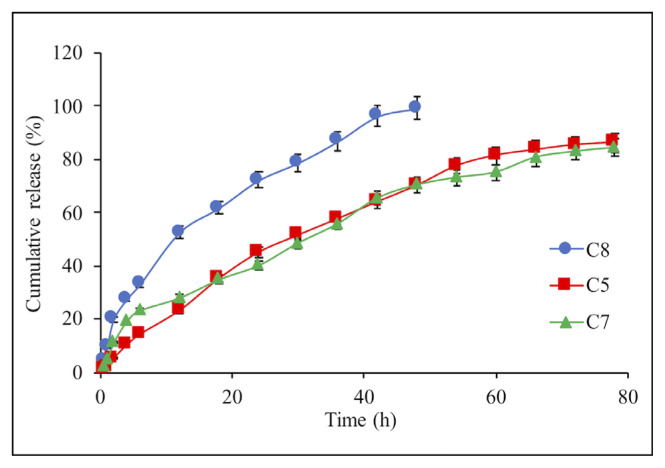
Effect of the CNF content on the IBR release from microspheres: C15 (–), C4 (0.05%), C5(0.1%), and C6 (0.2%). Each experiment was done three times in triplicates.

As seen in Figure 4, C15 microspheres showed the maximum primary burst release of IBR in the only early hours. The release profile of IBR from C15 realized fast and higher release rates about 7-fold of C6-coded composite microsphere within 12 h. An important reduction occurs in the primary burst release of IBR from microspheres by incrementing the CNF amount. This may be explained by the presence of CNF into composite resulted in a more extended pathway for migrating IBR from inside of composite into the release medium compared with the C15 microspheres; therefore, it prolongs the release time. Also, the attractive electrostatic interactions occur between the IBR and the CNFs oxidized as well as hydrogen bonding.

**Figure 5:**
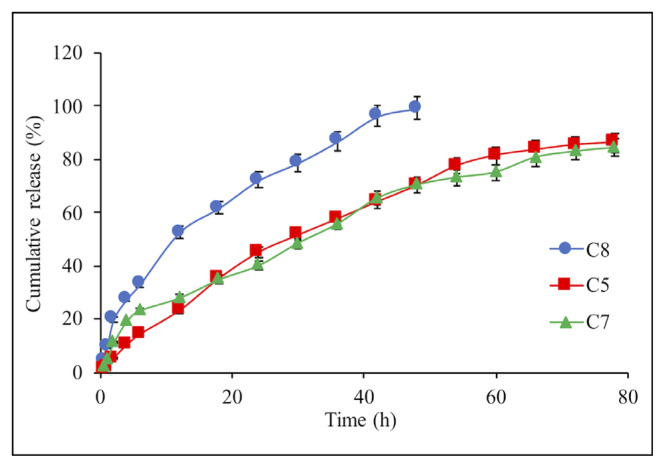
Effect of the drug concentration on the IBR release from composite microsphere: C8 (25%), C5 (10%), and C7 (20%). Each experiment was done three times in triplicates.

The release rate of IBR was studied with different concentrations (10%–25%) of the drug in the composite microspheres. As seen in Figure 5, there was no significant difference in drug release when the IBR concentration in composite structure increased from 10% to 20%. However, the increasing concentration to 25% caused a significant IBR release rate because of the increasing abundance of IBR amount in the composite network causing the higher diffusion rate of the drug [32].

**Figure 6:**
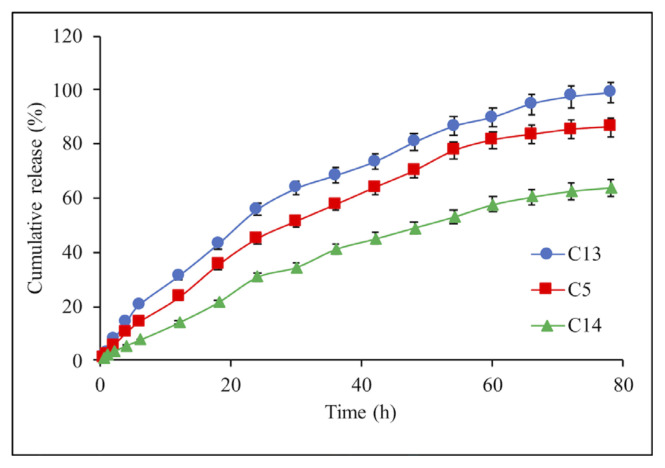
Effect of the chitosan concentrations on the IBR release from composite microspheres: C13 (1.5%), C5 (2%), and C14 (2.5%). Each experiment was done three times in triplicates.

The effect of chitosan concentration on IBR releasing profile was performed in the concentration ranges of 1.5–2.5% w/v (Figure 6). An obvious decrease is seen in the release rate of IBR with increasing the concentration of chitosan in the composite microspheres. Because of the increase in the polymer concentration, chitosan results in a thicker polymeric shell while increasing the crosslinking density, which causes the formation of a compact structure and, in the end, reduces drug release [29].

**Figure 7:**
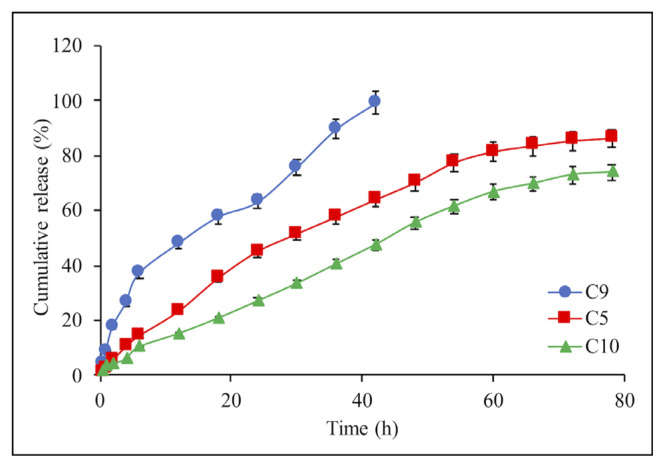
Effect of the TPP concentrations on the IBR release from composite microspheres: C9 (1%), C5 (2%), and C10(3%). Each experiment was done three times in triplicates.

The release rate of IBR decreased where the crosslinking agent TPP concentrations were increased from 1% w/v, 2% w/v, and 3% w/v in C9, C5, and C10-coded composite microspheres, respectively (Figure 7). Crosslinking was occurred through electrostatic forces between negative-charged phosphate groups of TPP and positive-charged amine groups in chitosan [33]. Thus, the crosslinking in the structure of the chitosan microspheres increases, and a rigid structure is formed that is responsible for more slow drug release. In other words, a less permeable denser matrix structure is formed for drug release [34].

**Figure 8:**
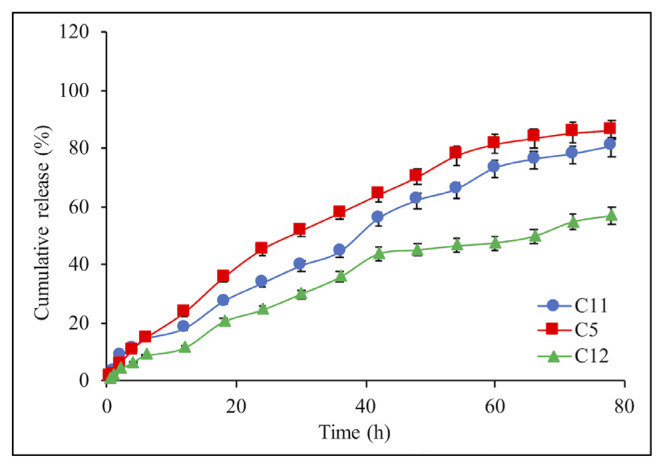
Effect of the crosslinking time on the IBR release from composite microspheres: C11 (60 min), C5 (30 min), and C12 (120 min). Each experiment was done three times in triplicates.

The effect of crosslinking time on the IBR release was carried out at 30, 60, and 120 min, and IBR release data was presented in Figure 8. The long crosslinking time leads to a decrease in drug release because more crosslinked and rigid TPP/chitosan matrices were obtained [35]. As observed in Figure 8, the composite microsphere prepared in TPP solution (%2, w/v) for 30 and 60 min showed faster release behavior than those of composite prepared for longer crosslinking time (120 min). When the crosslinking time increased from 30 min to 60 min, no significant difference was observed in terms of release rate while drug release for C12 was decreased with increasing the crosslinking time to 120 min. This is attributed to the fact that, a more crosslinked structure was formed with increasing the crosslinking time from 30 to 120 min, as was also reported in the literature [36]. Considering the stability of the structure and the encapsulation efficiency, it was decided that the 30 min crosslinking time was sufficient and admitted for further studies.

### 3.4. IBR release kinetics

The release data of IBR from composite microspheres was evaluated via fitting to three kinetic models, including zero order, first order, and Korsmeyer-Peppas. The IBR release kinetics parameters as a summary of release exponent (n) values and correlation coefficient were presented in Table 4. As seen in Table 4, the Korsmeyer-Peppas kinetic model was the best fitted model for the entire composite microsphere (R^2^ > 0.944). The exponent values for the release of IBR from the microsphere within 0.43–0.85 indicate a non-Fickian release performance. For both diffusion and swelling-controlled via swelling, the microspheres were the effective factors in controlling IBR release [16,37,38].

## 4. Conclusion

In this study, a CNF-incorporated chitosan-based drug delivery system was designed through dispersing oxidized CNF in the chitosan after crosslinking with TPP. The developed microspheres had a particle size between 0.58 and 1.16 mm. The obtained data demonstrated that the addition of CNF to the microsphere structure increased the encapsulation efficiency of the IBR from 39.87% to 87.75% and resulted in a more controlled and gradual release of the drug. C5-coded composite microspheres were determined to be the most suited formulation in terms of encapsulation efficiency and medication release rate (EE, 83.09%). The results indicated that the release data were fitted well to Korsmeyer-Peppas kinetic model (R^2^ > 0.944) and the release mechanism was anomalous diffusion (swelling-controlled behavior and diffusion), in which IBR could release at pH 6.8 within 78 h in a more controlled manner. Finally, the development of CNF based chitosan microsphere is a promising material to assure appropriate dosage use, safety, and improving drug efficiency.

## Figures and Tables

**Table 1:** Formulations of the IBR@CS/TPP/CNF microspheres.

Code	Ibrutinib (%w/w)	CNFs (%w/v)	TPP (%w/v)	pH of the external phase	Chitosan (%w/v)	Crosslinking time (min)
C1	10	0.05	1	5.0	2	30
C2	10	0.05	1	7.0	2	30
C3	10	0.05	1	8.8	2	30
C4	10	0.05	2	5.0	2	30
C5	10	0.1	2	5.0	2	30
C6	10	0.2	2	5.0	2	30
C7	20	0.1	2	5.0	2	30
C8	25	0.1	2	5.0	2	30
C9	10	0.1	1	5.0	2	30
C10	10	0.1	3	5.0	2	30
C11	10	0.1	2	5.0	2	60
C12	10	0.1	2	5.0	2	120
C13	10	0.1	2	5.0	1.5	30
C14	10	0.1	2	5.0	2.5	30
C15	10	-	2	5.0	2	30

**Table 2:** Particle size analysis of the formulations.

Formulation code	Arithmetic mean particle size (mm)	Formulation code	Arithmetic mean particle size (mm)
C1	1.04 ± 0.01	C9	0.88 ± 0.04
C2	1.14 ± 0.09	C10	1.06 ± 0.05
C3	1.10 ± 0.11	C11	0.94 ± 0.02
C4	1.02 ± 0.03	C12	0.96 ± 0.12
C5	0.90 ± 0.01	C13	0.58 ± 0.04
C6	1.08 ± 0.06	C14	1.16 ± 0.03
C7	0.94 ± 0.02	C15	0.94 ± 0.01
C8	0.84 ± 0.03	-	-

**Table 3:** Encapsulation efficiency of the formulations.

Formulation code	Encapsulation efficiency (EE, %)	Formulation code	Encapsulation efficiency (EE, %)
C1	49.89	C9	72.46
C2	31.55	C10	87.22
C3	17.80	C11	81.23
C4	81.60	C12	77.01
C5	83.09	C13	79.85
C6	84.31	C14	87.55
C7	83.58	C15	39.87
C8	86.10	-	-

**Table 4:** Release kinetics data for chitosan microspheres.

Formulation	Zero-order	First-order	Korsmeyer-Peppas	
	R^2^	R^2^	R^2^	n
C4	0.934	0.677	0.983	0.842
C5	0.936	0.663	0.990	0.812
C6	0.961	0.707	0.985	0.78
C7	0.929	0.626	0.944	0.758
C8	0.946	0.682	0.967	0.703
C9	0.953	0.668	0.962	0.741
C10	0.977	0.745	0.983	0.655
C11	0.949	0.684	0.961	0.761
C12	0.917	0.640	0.975	0.827
C13	0.941	0.636	0.968	0.845
C14	0.958	0.744	0.982	0.666
C15	0.887	0.632	0.957	0.762
